# Development and validation of patient diabetes knowledge questionnaire (PDKQ)

**DOI:** 10.1186/s40545-023-00631-3

**Published:** 2023-10-19

**Authors:** Phei Ching Lim, Retha Rajah, Yen Li Lim, Jason Lye Hin Kam, Te Ying Wong, Vivegananth Krishnanmurthi, Chee Tao Chang, Mun Teng Cheah, Nur Dayana Kamaruzzaman, Wee Toong Tan, Eng Seng Lee, Hadzliana Zainal

**Affiliations:** 1 Pharmacy Department, Hospital Pulau Pinang, George Town, Penang Malaysia; 2 School of Pharmaceutical Sciences, Universiti Sains Malaysia, Gelugor, Penang Malaysia; 3 Pharmacy Department, Hospital Seberang Jaya, Perai, Penang Malaysia; 4 Clinical Research Centre, Hospital Pulau Pinang, George Town, Penang Malaysia; 5 Clinical Research Centre, Hospital Raja Permaisuri Bainun, Ministry of Health Malaysia, Ipoh, Perak Malaysia; 6 School of Pharmacy, Monash University Malaysia, Subang Jaya, Selangor Malaysia

**Keywords:** Patient knowledge, Diabetes mellitus, Questionnaire, Development, Validation

## Abstract

**Background:**

Evaluation of diabetes knowledge plays a pivotal role in identifying and addressing patients’ knowledge gaps. The implementation of a standardized diabetes knowledge assessment tool is important to ensure consistent scoring and facilitating the development of effective and standardized education programs.

**Aim:**

To develop and validate a patient diabetes knowledge questionnaire (PDKQ) to assess knowledge of diabetes mellitus patients.

**Methods:**

The development of the PDKQ questionnaire involved three phases: item development, content validation, and reliability testing. In the item development phase, the initial draft of the PDKQ, comprising a multiple-choice answer questionnaire was developed. The content validation phase comprised two stages. Firstly, ten experts participated in the expert validation process, followed by face validation involving six patients. In the final phase, test–retest analysis was performed among diabetes mellitus patients to assess reliability.

**Results:**

The first draft of PDKQ consisted of 11 patient characteristics items and 37 items of multiple choices questions. During the expert validation, three items were eliminated due to low clarity, and an additional six items were removed as they were deemed irrelevant or unimportant. During the face validation, three patients' characteristic items and eight multiple-choice questions were excluded due to a content validity index of less than 0.83. In the test–retest phase, 36 subjects responded to 8 items pertaining to patients' characteristics and 20 multiple-choice questions. The test–retest analysis yielded an intraclass correlation coefficient of 0.88, indicating good reliability.

**Conclusion:**

The 20-item PDKQ is a reliable and robust tool in assessing the knowledge of diabetes mellitus patients in Malaysia. Its implementation allows standardized assessment of diabetic patients' knowledge levels, enabling targeted interventions to empower patients and optimize diabetes care practices.

## Introduction

Diabetes is one of the major public health concerns in the twenty-first century. Currently, half a billion people in the world are living with diabetes, and by 2030, it is estimated to rise to 643 millions adults aged 20–79 years [1]. Similarly, diabetes prevalence increased remarkably among the Asian population in the last few decades and is more likely in the younger age group as compared to the Western population [2]. In Malaysia, National Health and Morbidity Survey 2019 (NHMS 2019) reported that one in five people aged 18 years and above suffered from diabetes [3]. The alarming and projected increase in these numbers calls for the urgent implementation of coordinated strategies to tackle this disease.

Management of diabetes is complex, multi-faceted and needs high patient involvement to perform self-care activities, self-monitoring, and medication adherence. Patient education is vital to diabetes care as it empowers patients with essential knowledge and skills necessary for appropriate self-management [4]. A previous study revealed that pharmacist-led educational intervention significantly improved glycemic control [5]. Assessment of diabetes knowledge is essential for healthcare providers in identifying the patients’ knowledge gap and providing effective individualized education programs. Patients’ knowledge of diabetes can also be used as one of the outcome measures to assess the effectiveness of the educational intervention [6].

Several instruments were developed to assess diabetes knowledge that includes the Michigan Diabetes Knowledge Tool (MDKT) [7], Diabetic Knowledge Questionnaire (DKQ) [8, 9], Diabetes Knowledge Assessment (DKN) scale [10], the Ped-Carb Quiz (PCQ) [11] and the Diabetic Numeracy Test (DNT) [12]. These instruments were developed and validated in the United States [7–9, 11, 12] and Australia [10]. They were varied in assessment objectives. The MDKT was the earliest instrument developed to assess knowledge on general issues and insulin use [7]. The DKQ assessed general diabetes knowledge [8, 9], while the PCQ assessed carbohydrate food recognition, carbohydrate food counting, and the incorporation of carbohydrate counting in calculating insulin dose [11]. Meanwhile, the DNT measured numeracy skills for diabetes, such as food label interpretation, insulin dose calculation based on blood glucose level, and carbohydrate corrections [12]. These instruments have been adapted and adopted in other countries.

In Malaysia, validated and translated MDKT had been utilized in two studies conducted in diabetes clinics [13] and in a few pharmacies in district areas using convenient sampling to assess the knowledge of type 2 diabetes patients [14]. Both studies used a brief 14-item MDKT instead of the complete 23-item MDKT [7], and the scoring was based on the number of correct items. On the other hand, two studies utilized different knowledge assessment tools scored based on percentage. One study, conducted in the district area of Seremban, utilized a 41-item questionnaire [15] which included items from the questionnaires of Wee et al. [16] and Tham et al. [17]. Meanwhile, another study conducted in a specialized diabetes clinic in Kelantan [18] used a 15-item DKN questionnaire.

A wide variety of adapted tools were used in Malaysia, leading to variations in the scoring methods. The variation in the assessment tools could result in inconsistency in result reporting and pose challenges when comparing studies. Therefore, having a reliable and validated instrument to assess diabetes knowledge among patients with diabetes mellitus in Malaysia is essential to meet the specific educational needs of this population. The current study aimed to report the development and validation of a new diabetes knowledge questionnaire, the patient diabetes knowledge questionnaire (PDKQ).

## Methods

The development of PDKQ questionnaire consisted of three phases: item development, content validation, and reliability test. This study was registered with the National Medical Research Register of Malaysia (NMRR ID: NMRR-20-1844-55868) and approved by Medical Research Ethics Committee, Malaysia.

### Phase 1: item development

The item development phase consisted of three steps: item generation, item deduction, and questionnaire formatting.

The item generation process involved a systematic and vigorous literature search of existing published English language diabetes knowledge tools. The systematic review was conducted in accordance with the Preferred Reporting Items for Systematic Reviews and Meta-Analysis (PRISMA) using six electronic databases: CINAHL, Medline, Google Scholar, PubMed, Sage Journals, and Science Direct. This systematic review was published in the Review of Diabetic Studies in 2021 [19]. This review included seven studies that comprised 99 items and divided into eight domains: 29 questions on disease-specific, 21 questions on nutrition, 18 questions on treatment, 12 questions on adverse effects, 9 questions on monitoring, 5 questions on physical activity, 4 questions on risk factors, and 1 question on foot care [19]. The questionnaires utilized were (1) translated and validated MDKT; (2) a questionnaire from Wee et al. and Tham et al.; (3) translated DKN; (4) translated and validated American Association of Clinical Endocrinologists (AACE); and (5) validated Theptarin Diabetes questionnaire. Items for the questionnaire were generated from the findings of the systematic review. Some items were developed based on the locally available Diabetes Medication Therapy Adherence Clinic protocol by the Ministry of Health, Malaysia (16 items) [20] and additional domain related to Ramadan (4 items).

The item deduction step involved six members of expert in three meetings. The members consisted of pharmacists who were involved in the management of diabetes patients in the ward, ambulatory care, and counseling. A nominal technique was utilized to identify the domains and prioritize the key areas of diabetes-related knowledge. Each item in the key area was evaluated in terms of relevance, representativeness, and value to educate diabetes mellitus patients. Every item was voted and comments were made to modify, keep or remove the items. Items that consistently received votes for removal were eliminated, and modifications were made to the commented items. Following extensive discussions, the reranking process was carried out until a consensus was reached, ensuring that no further changes were necessary in the questionnaire.

The first draft of PDKQ was formatted and presented as questions with multiple-choice answers, enabling the identification of specific knowledge gaps for personalized and targeted education.

### Phase 2: content validation

#### Expert validation

The first draft of PDKQ was sent to experts selected from different states in Malaysia for content validity. The ten experts included in this study comprised consultant endocrinologists, family medicine specialists, and pharmacists who represented different states in the Working Committee of Clinical Pharmacy specializing in diabetes. The expert validation was conducted through a non-face-to-face approach. The content validation form, expert information sheet, clear information, and informed consent form were emailed to the experts. The experts evaluated the relevancy, importance, and clarity of each item for its corresponding construct on a 4-point scale (4 being either very relevant, very important, or highest clarity, whereas 1 being either not relevant, not important, or no clarity). The experts were asked to provide feedback on the questions to improve the quality of the items.

Content validity ratio (CVR) was calculated for items clarity based on the formula, CVR = (Ne − *N*/2)/(*N*/2), where Ne is the number of experts indicated ‘essential’ and *N* is the total number of experts. The content validity ratio was between 1 and − 1. A higher score indicated better agreement between the members of the experts on the necessity of an item in the questionnaire. Based on the Lawshe table, the items were considered acceptable if they achieved a CVR value of at least 0.62 [21]. The content validity index (I-CVI) and scale-level content validity index were calculated to measure proportional agreement. The Scale-level Content Validity Index (S-CVI/Ave) was computed on average to indicate content validity. An I-CVI of 0.78 or higher was considered to have excellent content validity, given the involvement of 10 experts [22]. For the scale-level content validity index, S-CVI/Ave of 0.9 or higher indicated acceptable content validity [23]. The second draft of PDKQ was provided after modifying and eliminating the items based on feedbacks from the experts.

#### Face validation

Potential subjects were recruited for face validation to test the appropriateness of the questionnaire in terms of construct, language clarity, readability, and feasibility. Patients of either gender aged more than 18 years with diabetes mellitus and who understood English were included, whereas patients who had cognitive impairment or psychiatry-associated illnesses such as dementia, Alzheimer, schizophrenia, and mania were excluded. All subjects provided written informed consent.

A cognitive interview was conducted with six patients in two rounds. The second draft of the PDKQ, along with the demographic data, was administered to the subjects. Confusing questions were identified and modified to improve clarity or to be removed. Problematic, irrelevant, or unimportant questions and demographic item were identified and removed. The order of questions was rearranged. The subjects graded the relevancy and importance of each item on a 4-point scale (4 being either very relevant or very important, whereas 1 is either not relevant or not important). Content validity (I-CVI) was calculated, and a score of at least 0.83 was considered excellent validity [24]. The outcomes of the cognitive interviews were recorded. Modifications were made to grammar and word choice. Items were dropped if they lacked clarity, relevance, and importance to produce the third draft of PDKQ.

### Phase 3: reliability test

A pilot test was conducted for test–retest analysis. Patients aged 18 years and above with diabetes mellitus who understood English were recruited and consented to participate. Patients who were unwilling to consent or with cognitive impairment or psychiatric disorders were excluded. Considering the dropout rate of 20%, 36 patients were recruited [25]. The patients completed the questionnaire on Day 1 and then returned to re-administer the same questionnaire after 14 days. The correct answer for the multiple-choice questionnaire was scored as 1, while the incorrect answer was scored as 0. The total score of the first and second responses were measured.

Demographic data such as age, duration of diabetes, smoking, underlying comorbidities, and education level of the patients were collected. Data were analyzed using SPSS 22.0 (IBM Corp., New York, USA). Intraclass correlation coefficient analysis was performed to assess the test–retest reliability of the responses. Spearman rank correlation coefficient was used to analyze overall score stability. An intraclass coefficient value greater than 0.90 indicated excellent reliability, while a value less than 0.5 indicated poor reliability. A value between 0.5 and 0.75 indicated moderate reliability, and a value between 0.75 and 0.9 indicated good reliability [26].

## Results

**Fig. 1:**
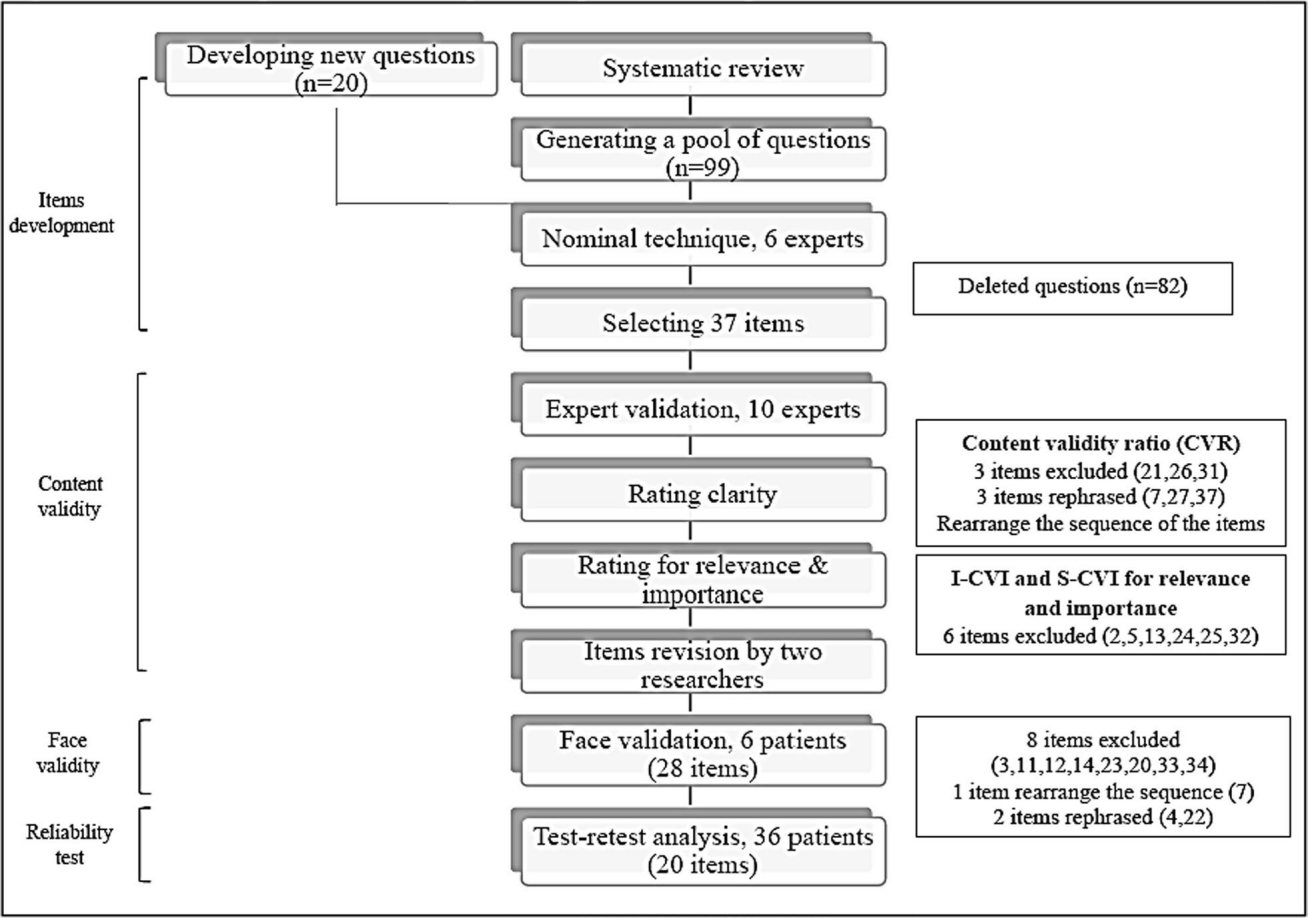
Flow diagram describing the steps to develop and validate PDKQ

The flow of the development and validation of PDKQ is summarized in Fig. 1.

### Items development

The initial pool of questions comprised 119 items and nine domains. During the nominal technique, 69% of the items were removed as duplication and did not reach the consensus. The experts also decided to exclude two domains from the questionnaire: foot care and Ramadan. The domain of foot care was not part of the DMTAC protocol, while the domain of Ramadan was applicable only to Muslim patients.

Subsequently, 37 items were retained (Fig. 1). Seventeen items (45.9%) were modified to enhance understandability in the target population. The first draft of PDKQ consisted of two parts. The first part consisted of 11 patient characteristics items, and the second consisted of 37 items with multiple-choice questions.

### Expert validation

During the expert validation process, three items (8.1%) were excluded due to low clarity, with CVR values less than 0.6 (Table 1). However, items 7, 27, and 37 were rephrased and retained (Table 2), as they achieved a CVR of more than 0.6 (Table 1). Furthermore, six items (16.2%) were eliminated as they were deemed irrelevant or unimportant, with I-CVI values less than 0.78 (Table 3). The S-CVI/Ave scores of 0.92 and 0.94 for relevancy and importance of the remaining items indicated that they were effective operationalizations of the underlying construct.

**Table 1:** Content validity ratio (CVR) to assess the clarity (expert validation)

Items	Ne	CVR	Interpretation
1	10	1	Remained
2	10	1	Remained
3	10	1	Remained
4	10	1	Remained
5	10	1	Remained
6	10	1	Remained
7	8	0.6	Remained
8	10	1	Remained
9	10	1	Remained
10	10	1	Remained
11	10	1	Remained
12	10	1	Remained
13	9	0.8	Remained
14	10	1	Remained
15	9	0.8	Remained
16	10	1	Remained
17	10	1	Remained
18	9	0.8	Remained
19	9	0.8	Remained
20	10	1	Remained
21	5	0	Eliminated
22	10	1	Remained
23	10	1	Remained
24	10	1	Remained
25	9	0.8	Remained
26	7	0.2	Eliminated
27	8	0.6	Remained
28	10	1	Remained
29	10	1	Remained
30	10	1	Remained
31	7	0.2	Eliminated
32	9	0.8	Remained
33	10	1	Remained
34	10	1	Remained
35	10	1	Remained
36	9	0.8	Remained
37	8	0.6	Remained

**Table 2:** Wording problems in PDKQ

Question no.	Original wording	Change
Expert validation		
7	Can be cured	All patients can be cured
	Is a progressive disease	Is a progressive disease that can lead to a lot of complications
27	Potato	Biscuits
37	Random blood sugar	Blood sugar immediately after meal
Face validation		
1, 4, 6, 8, 9, 19, 29, 35, 36, 37	Glucose	Sugar
4	Risk factors of type 2 diabetes are as follows EXCEPT	Risk factors of type 2 diabetes are as follows
	Obesity	Overweight
	Blurred vision	All of the above
22	Stop taking	Throw away

**Table 3:** Content validity index (I-CVI) and scale content validity index (S-CVI) for item relevancy and importance (expert validation)

Items	Relevancy				Importance			
	Relevant (rating 3 or 4)	Not relevant (rating 1 or 2)	I-CVI	Interpretation	Important (rating 3 or 4)	Not important (rating 1 or 2)	I-CVI	Interpretation
1	10	0	1	Relevant	10	0	1	Important
2	7	3	0.7	Eliminated	7	3	0.7	Eliminated
3	10	0	1	Relevant	10	0	1	Important
4	9	1	0.9	Relevant	9	1	0.9	Important
5	7	3	0.7	Eliminated	8	2	0.8	To revise
6	9	1	0.9	Relevant	9	1	0.9	Important
7	10	0	1	Relevant	9	1	0.9	Important
8	10	0	1	Relevant	10	0	1	Important
9	10	0	1	Relevant	10	0	1	Important
10	10	0	1	Relevant	10	0	1	Important
11	10	0	1	Relevant	10	0	1	Important
12	10	0	1	Relevant	10	0	1	Important
13	7	3	0.7	Eliminated	8	2	0.8	To revise
14	10	0	1	Relevant	10	0	1	Important
15	10	0	1	Relevant	10	0	1	Important
16	10	0	1	Relevant	10	0	1	Important
17	10	0	1	Relevant	10	0	1	Important
18	8	2	0.8	To revise	8	2	0.8	To revise
19	9	1	0.9	Relevant	10	0	1	Important
20	10	0	1	Relevant	10	0	1	Important
21	10	0	1	Relevant	10	0	1	Important
22	10	0	1	Relevant	10	0	1	Important
23	10	0	1	Relevant	10	0	1	Important
24	5	5	0.5	Eliminated	7	3	0.7	Eliminated
25	7	3	0.7	Eliminated	7	3	0.7	Eliminated
26	9	1	0.9	Relevant	10	0	1	Important
27	10	0	1	Relevant	10	0	1	Important
28	10	0	1	Relevant	10	0	1	Important
29	10	0	1	Relevant	10	0	1	Important
30	10	0	1	Relevant	10	0	1	Important
31	10	0	1	Relevant	10	0	1	Important
32	7	3	0.7	Eliminated	6	4	0.6	Eliminated
33	10	0	1	Relevant	10	0	1	Important
34	9	1	0.9	Relevant	9	1	0.9	Important
35	10	0	1	Relevant	10	0	1	Important
36	10	0	1	Relevant	10	0	1	Important
37	9	1	0.9	Relevant	9	1	0.9	Important

### Face validity

Six patients (66.7% female, 50.0% Malay, 33.3% Chinese, 16.7% Indian) were included for face validation. Two patients commented that the questionnaire contained too many questions. Eight items (21.6%) were then eliminated from the questionnaire, as their I-CVI scores were less than 0.83 (Table 4).

**Table 4:** Content validity index (I-CVI) for item relevancy and importance (face validity)

Items	Relevancy				Importance			
	Relevant (rating 3 or 4)	Not relevant (rating 1 or 2)	I-CVI	Interpretation	Important (rating 3 or 4)	Not important (rating 1 or 2)	I-CVI	Interpretation
1	6	0	1	Relevant	6	0	1	Important
3	4	2	0.67	Eliminated	5	1	0.83	Important
4	6	0	1	Relevant	6	0	1	Important
6	6	0	1	Relevant	6	0	1	Important
7	6	0	1	Relevant	5	1	0.83	Important
8	6	0	1	Relevant	6	0	1	Important
9	6	0	1	Relevant	6	0	1	Important
10	6	0	1	Relevant	6	0	1	Important
11	5	1	0.83	Relevant	4	2	0.67	Eliminated
12	5	1	0.83	Relevant	4	2	0.67	Eliminated
14	4	2	0.67	Eliminated	2	4	0.33	Eliminated
15	5	1	0.83	Relevant	6	0	1	Important
16	6	0	1	Relevant	6	0	1	Important
17	6	0	1	Relevant	6	0	1	Important
18	6	0	1	Relevant	6	0	1	Important
19	6	0	1	Relevant	6	0	1	Important
20	6	0	1	Relevant	6	0	1	Important
22	5	1	0.83	Relevant	6	0	1	Important
23	5	1	0.83	Relevant	4	2	0.67	Eliminated
27	6	0	1	Relevant	6	0	1	Important
28	6	0	1	Relevant	6	0	1	Important
29	6	0	1	Relevant	5	1	0.83	Important
30	4	2	0.67	Eliminated	4	2	0.67	Eliminated
33	4	2	0.67	Eliminated	4	2	0.67	Eliminated
34	5	1	0.83	Relevant	4	2	0.67	Eliminated
35	6	0	1	Relevant	6	0	1	Important
36	5	1	0.83	Relevant	6	0	1	Important
37	6	0	1	Relevant	6	0	1	Important

Additionally, the wordings in few items were changed to improved the clarity of the items (Table 2). During the face validation interviews, patients expressed that their occupation sector, marital status, and monthly income were not relevant to their disease or knowledge. Therefore, these three items on patients' characteristics were deleted, and only eight items on patients' characteristics were included in the third draft of PDKQ.

### Test–retest reliability

The third draft of PDKQ consisted of two parts, with the first part consisting of 8 items on patients' characteristics and 20 items of multiple-choice questions (Fig. 1). Thirty-six patients consented to participate in the reliability test and the demographic data are presented in Table 5. The analysis of the internal test–retest score demonstrated good reliability with the intraclass correlation coefficient of 0.88 (95% CI 0.78,0.94), p < 0.001. The overall test–retest score demonstrated a strong correlation, with a Spearman's rho of 0.76. These results supported the reliability of 20-item PDKQ (Table 6). Item 15 and 37 had the highest percentage of incorrect answers, with more than 50% of the subjects providing incorrect responses.

**Table 5:** Demographic data of test–retest analysis (*n* = 36)

Characteristics	Number of patients, (%)
Age^a^	49.47 ± 16.35
Duration of diabetes mellitus^a^	11.44 ± 8.21
Gender	
Female Male	16 (44.4) 20 (55.6)
Ethnicity	
Malay Chinese Indian Other	12 (33.3) 18 (83.3) 5 (13.9) 1 (2.8)
Education	
No formal education Primary education Secondary education Tertiary education	1 (2.8) 2 (5.6) 21 (58.3) 12 (33.3)
Family history of diabetes	26 (72.2)
Smoking	
Yes No Ex-smoker	1 (2.8) 32 (88.9) 3 (8.3)
Comorbids	
Hypertension Dyslipidemia Cardiovascular disease Kidney disease Stroke Retinopathy Neuropathy	21 (58.3) 20 (55.6) 5 (13.9) 2 (5.6) 2 (5.6) 9 (25.0) 10 (27.8)
Others	9 (25.0)
Score test^b^	16 (4)
Score retest^b^	16 (4)

^a^Data presented in mean ± SD years

^b^Data presented in median (IQR)

**Table 6:** PDKQ (20-item questionnaire)

Domains	Questions (*n*)
Disease specific	4
Treatment	5
Nutrition	2
Adverse effects	5
Monitoring	2
Risk factors	1
Physical activities	1

## Discussion

The PDKQ represents a unique questionnaire specifically crafted to assess patients' knowledge of diabetes in Malaysia. Throughout its development, the PDKQ has undergone rigorous testing phases, ensuring its validity and reliability. The final 20-item questionnaire demonstrated strong reliability with an Intraclass Correlation Coefficient (ICC) of 0.88, indicating that the measures used in this study generated consistent and stable results. These robust findings solidify the PDKQ as a valid and reproducible instrument for assessing diabetes knowledge among patients in Malaysia.

During the reliability test, it was observed that item number 15 had the highest number of incorrect responses from patients. This item was related to knowledge about the storage of insulin that had been used. Subjects might be confused about the word “used” insulin, as the storage condition for both “used” and “un-used” insulin was different. The insulin needs to be stored in the refrigerator at 2–8 °C. On the other hand, the insulin in use could last up to 4 weeks at room temperature of not more than 28 °C and away from sunlight [27]. The phrase "has been used" should be rephrased to “in use” for better understanding. Similarly, in a previous study, it was reported that 51.2% of diabetic pilgrims from 22 countries during the 2019 Hajj were not aware of the appropriate duration of insulin at room temperature, despite more than 90% being aware that unused insulin should be stored in a refrigerator [28]. Insulin is a labile protein susceptible to elevated temperatures, vigorous agitation, and exposure to sunlight. Therefore, adhering to proper storage protocols is imperative to maintain its efficacy and potency. The improper storage of insulin can have serious consequences, potentially leading to therapy failure as the potency of insulin decreases. Research has shown that inadequate knowledge regarding insulin storage is associated with poor glycemic control [27]. Patient awareness of proper insulin storage is essential to ensure the effectiveness of diabetes management.

Meanwhile, item number 37 evaluated the subjects' knowledge of blood glucose monitoring, and the findings revealed that their understanding of targets for achieving good blood glucose control was inappropriate and quite concerning. Adequate knowledge of glycemic control targets has been shown to positively impact glycemic control, medication adherence, and overall self-management of the disease [29]. This finding underscores the importance of providing education on therapeutic targets to empower patients to take charge of their self-management.

The Michigan Diabetes Knowledge Test (MDKT) is a well-established and internationally recognized tool for reliably assessing the knowledge of diabetes patients [7]. Having been developed over two decades ago in the United States, the Michigan Diabetes Knowledge Test (MDKT) may not align perfectly with the local context in Malaysia and the contemporary approaches to diabetes management. The translated Malay version of the Michigan Diabetes Knowledge Test (MDKT) comprised 14 items [13], whereas the PDKQ developed in this study comprised 20 items. While generating the pool of questions, the PDKQ was thoughtfully organized into seven domains: disease-specific, treatment, nutrition, physical exercise, monitoring, adverse events and risk factors. On the other hand, the translated version of the MDKT only covered five domains, which included disease-specific, nutrition, physical exercise, monitoring, and foot care [13]. The inclusion of additional domains in the PDKQ provides a more comprehensive assessment of diabetes knowledge to facilitate diabetes management and care.

Although translated and validated, certain questions in MDKT might not be suitable in Malaysia diabetes population, particularly the nutrition part. Similarly, questions related to carbohydrate in the validated American Association of Clinical Endocrinologists (AACE) questionnaire, might not align well with the Malaysian context [30]. As the Malaysian primary carbohydrate source is rice-based [31], while the American diet predominantly comprises corn and wheat [32], some food items mentioned in the MDKT and AACE, such as Swiss cheese, may not be as familiar to the local population. Therefore, to ensure the PDKQ's appropriateness for Malaysia, we carefully considered cultural and dietary differences, crafting questions that better resonate with local lifestyle and food choices. This approach enhances the questionnaire's relevance in accurately assessing diabetes knowledge among Malaysian patients, facilitating effective education interventions. While ensuring the PDKQ appropriateness to the Malaysian diet, it may be generalized to other centers and countries in Asia especially South East Asia, considering their similar staple diet being rice [33]. As a whole, the remaining items in the PKDQ also covered a broad context allowing it to be generalizable to most populations with diabetes.

The length of a questionnaire can significantly impact the quality of responses provided by participants. Lengthy questionnaires have been associated with various challenges, such as decreased response rates, response fatigue, and reduced participant engagement, which in turn lead to diminished attention and comprehension of the questions [34, 35]. Consequently, this could compromise the reliability and accuracy of the data collected. An ideal questionnaire typically consists of less than 30 questions and should be feasible to complete within approximately 30 min [35]. A similar local study conducted by Ding et al. utilized a questionnaire comprising 41 questions, which resulted in 15% of incomplete responses [15]. In contrast, the PDKQ developed in this study consisted of 20 items. This deliberate decision to reduce the number of questions aimed to enhance the quality of responses.

This study had few limitations. While the expert validation involved experts from various regions in Malaysia, it is important to note that the subjects for the study were recruited from a single center. The study site was a tertiary public hospital in an urban area, where patients may have had a longer duration of diabetes and more severe disease progression compared to those in primary care settings. The development of the PDKQ was conducted in English, which might have resulted in bias in patient selection. This was because not every patient could understand English, particularly elderly and less educated patients. It is important to acknowledge that the sample size in this study was relatively small, which may limit the generalizability of the findings. Nevertheless, PDKQ consisted of broad questions and referred to protocol by Ministry of Health Malaysia [20] which were used by all the public health facilities in Malaysia. Hence, PDKQ can be used in other healthcare facilities. It is worth noting that the study was conducted with rigor and followed a systematic approach to questionnaire development and validation.

This study represents a significant milestone in the development of a validated diabetes knowledge assessment tool for diabetic patients. The validation of the PDKQ in English serves as a foundation for its future translation into Malay and subsequent validation with more extensive and diverse populations from multiple centers.

## Conclusion

The 20-item PDKQ demonstrates high reliability and robustness in assessing the knowledge of diabetes mellitus patients in Malaysia. By implementing the PDKQ, healthcare professionals can assess patients' knowledge levels in a standardized manner, facilitating the design of targeted interventions to empower patients and optimize diabetes care practices.

## Data Availability

The datasets generated and/or analyzed during the current study are not publicly available due to confidentiality of patients, but are available from the corresponding author on reasonable request.
